# Survival outcomes in node-positive non-metastatic bladder cancer: An analysis of the national cancer database

**DOI:** 10.1080/2090598X.2022.2077001

**Published:** 2022-05-20

**Authors:** Amr A Elbakry, Tyler Trump, Christopher Ferari, Malcom D Mattes, Adam Luchey

**Affiliations:** a Department of Urology, West Virginia University, Morgantown, WV, USA; b Department of Urology, Indiana University, Indianapolis, IN, USA; c Department of Radiation Oncology, Rutgers Robert Wood Johnson, New Brunswick, NJ, USA

**Keywords:** bladder cancer, radiation therapy, radical cystectomy, national cancer database

## Abstract

**Introduction:**

Clinically node-positive non-metastatic bladder cancer (cN+) has been the target of several studies aiming to establish a standard of care for this population. Limited studies have shown a survival benefit for various multimodal therapy approaches. The role of radiation therapy has not been well established. Our study aims to study the trends of the reported treatment options offered to patients with cN+ bladder cancer in a national database and to evaluate the effect of various treatments, including radiation, on survival.

**Methods:**

The National Cancer Database (NCDB) was used to identify cN+ bladder cancer patients who received chemotherapy alone or in combination with radical cystectomy (RC) or radiotherapy. 3,481 patients were included and divided into 4 groups: chemotherapy only, chemotherapy and RC, chemotherapy and radiation therapy, and chemotherapy, RC, and radiation therapy. Demographic data was compared using ANOVA for continuous variables, and Chi-square for categorical variables. Multivariable analysis was done to compare groups using a multinomial logistic regression model. Kaplan-Meier test was used for survival analysis and Cox-Regression was used for multivariable survival analysis.

**Results:**

Patients undergoing RC were significantly younger (*P* <0.001). There was a significant difference between the groups regarding racial distribution, facility-type and insurance status. There was no difference in gender, Charlson\Deyo score, financial or educational status. Patients who underwent combination therapy with chemotherapy and RC were found to have the longest median survival time at 27 months. Multivariable analysis showed that final treatment, age, sex, Charlson\Deyo comorbidity score, TNM edition and facility-type were significant survival predictors. Race, insurance and financial status failed to maintain significance. There was no survival difference between the chemotherapy group and chemo-radiotherapy group.

**Conclusions:**

The combination of surgery and chemotherapy achieves statistically significant superior survival in cN+ bladder cancer. Adding radiotherapy to chemotherapy did not improve survival in this group of patients.

**Abbreviations:**

(cN+): Clinically node-positive non-metastatic, (MIBC): Muscle invasive bladder cancer, (NCDB): National Cancer Database, (NAC): Neoadjuvant chemotherapy, (RC): Radical Cystectomy

## Introduction

Bladder cancer represents the sixth most common cancer in the United States. It is estimated that there will be over 80,000 new cases of bladder cancer annually, with nearly 18,000 estimated deaths from this disease [1]. Muscle invasive bladder cancer (MIBC) represents an aggressive subset of bladder cancer with an increased propensity for metastasis, but when clinically localized, remains curable [2]. Clinical trials involving treatment of MIBC are abundant in the literature. Neoadjuvant chemotherapy (NAC) with a cisplatin-based regimen followed by radical cystectomy (RC) remains the gold standard treatment for clinically localized MIBC [3]. Patients who present with distant metastatic disease also receive a therapeutic benefit from chemotherapy, but rarely achieve cure. Despite the robust evidence surrounding management of both clinically localized and metastatic bladder cancer, there remains a paucity of information regarding management of patients with disease which has spread only to regional lymph nodes (cN+).

Those with cN+ disease have been excluded from large trials of neoadjuvant chemotherapy as they are historically grouped in with distant metastatic disease [4,5] Several smaller retrospective analyses have been performed, showing some promise for specific treatment modalities in this population [3,6]. The consensus among these is that induction chemotherapy followed by RC can provide a survival benefit in cN+ patients. One large, retrospective cohort study examined cN+ patients from the National Cancer Database (NCDB) who underwent chemotherapy, radical cystectomy, or a combination of these modalities, finding the highest 5-year OS with preoperative chemotherapy followed by RC. This patient cohort did not include those who underwent radiotherapy [7].

Despite these studies of various sizes and generalizability, significant gaps still exist regarding optimal treatment sequence and approach in cN+ bladder cancer patients. There is a relative paucity of studies that include radiotherapy in the discussion of treatment modality in this population. This study aims to use the NCDB to evaluate the effect of various combinations of treatment modality, including radiation therapy, on long-term survival in a large cohort of cN+ bladder cancer patients.

In this study, we are aiming to evaluate long-term survival with different treatment options in a large cohort of cN+ urothelial carcinoma. Also, we studied the trends of treatment options provided and the effect of different demographic and social factors on those trends.

## Methods

Data for this study was derived from the NCDB. The NCDB is a large national database comprising information from over 1500 hospitals created through collaborative efforts between the American College of Surgeons and the American Cancer Society. It is cited that the database comprises roughly 70% of newly diagnosed cancer cases within the United States [8]. All patient information from this database has been deidentified. Given the nature of the patient information collected the study was exempt from Institutional Review Board approval.

Using TNM staging from the American Joint Committee on Cancer we identified 6971 patients with cTanyN1-3M0 urothelial bladder cancer between the years 2004 and 2015. TNM staging was updated from the sixth to seventh edition in 2010; therefore, prior to 1 January 2010, the sixth edition was used for classification and following 1 January 2010, the seventh edition was used. Patients were excluded due to any of the following criteria: missing treatment data, missing demographic data, missing follow-up data, and treatment regimen not included in the study groups listed in the following paragraph. Data on the histological type of the tumor is limited in the database with no differentiation between different variants, so we did not exclude patients based on the tumor histology or histological variant type.

**Figure 1.**
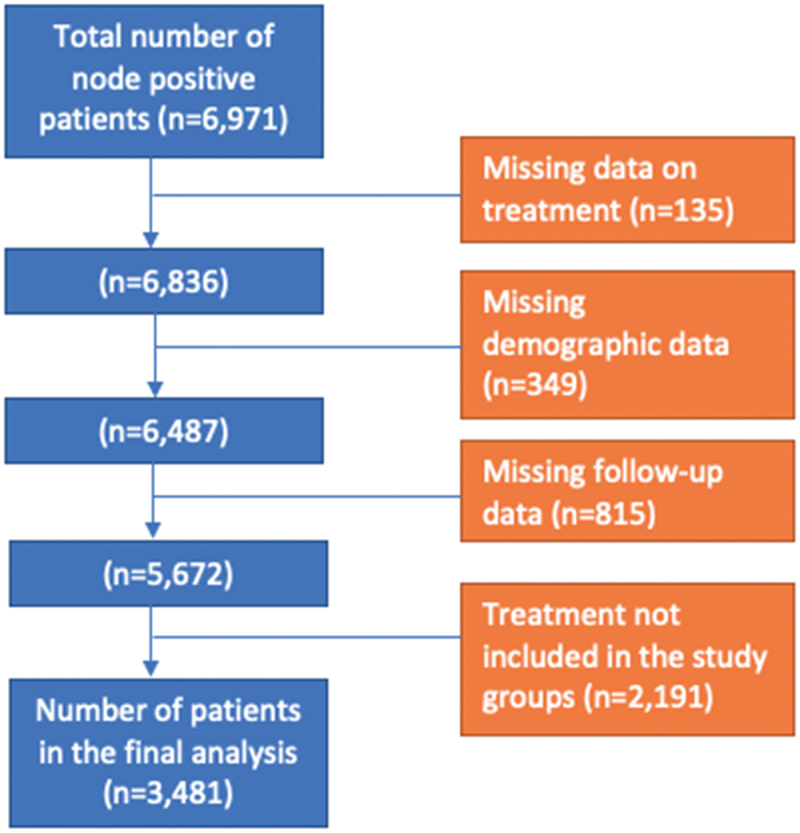
Flow chart of patients included in the analysis.

Following patient exclusions, we identified 3481 patients eligible for analysis (Figure 1). Patients were then grouped into 4 categories based on the treatment regimen they were provided. These categories were: chemotherapy only, chemotherapy and radical cystectomy, chemotherapy and radiation therapy, and chemotherapy, radical cystectomy, and radiation therapy. Information regarding chemotherapy and radiation timing and regimen/dose are not readily ascertained from the dataset provided.

We included several patient characteristics to further analyze the effect of treatment type received on survival while adjusting for confounding. These patient characteristics are summarized in Table 1 below and include: age, gender, race, comorbidities (as Charlson-Deyo scores), insurance status, median household income, education level, and facility-type at which treatment was conducted.

**Table 1.** Demographic and treatment data.

Variables	Group 1 Chemotherapy only	Group 2 RC + Chemotherapy	Group 3 Chemo-Radiotherapy	Group 4 RC + Chemo-Radiotherapy	P value
No.	1312 (37.7%)	1316 (37.8%)	726 (20.9%)	127 (3.6%)	
**Patient demographics**					
Age, mean (SD)	67 (11)	64 (9.7)	69 (11.7)	64 (10.2)	**<0.01**
Male, n. (%)	954 (72.7%)	945 (71.8%)	508 (70%)	86 (67.7%)	0.43
White, n. (%)	1167 (88.9%)	1210 (91.9%)	635 (87.5%)	117 (92.1%)	**0.01**
**Charlson\Deyo score, n. (%)**					0.54
0	977 (74.5%)	978 (74.3%)	562 (77.4%)	97 (76.4%)	
1	247 (18.8%)	265 (20.1%)	124 (17.1%)	23 (18.1%)	
≥2	88 (6.7%)	73 (5.5%)	40 (5.5%)	7 (5.5%)	
**Primary payer n. (%)**					**<0.01**
uninsured	60 (43.2%)	48 (34.5%)	26 (18.7%)	5 (3.6%)	
private	411 (34.9%)	560 (47.5%)	167 (14.2%)	40 (3.4%)	
Medicaid	104 (39.8%)	92 (35.3%)	54 (20.7%)	11 (4.2%)	
Medicare	724 (39%)	597 (32.1%)	467 (25.2%)	69 (3.7%)	
Other government	13 (28.3%)	19 (41.3%)	12 (26.1%)	2 (4.3%)	
**Median household income, n. (%)**					0.08
<38,000	235 (41.8%)	185 (32.9%)	119 (21.1%)	23 (4.1%)	
38,000–47,999	329 (36.6%)	333 (37.1%)	198 (22.1%)	37 (4.1%)	
48,000–62,999	382 (39%)	366 (37.3%)	197 (20.1%)	34 (3.4%)	
>63,000	366 (35%)	432 (42%)	212 (20%)	33 (3%)	
**No. high school diploma, n. (%)**					0.06
>21%	222 (40.7%)	187 (34.2%)	120 (22%)	17 (3.1%)	
13%-20.9%	342 (37.2%)	346 (37.7%)	204 (22.2%)	27 (2.9%)	
7%-12.9%	456 (37.1%)	455 (37%)	266 (21.7%)	51 (4.2%)	
<7%	292 (37%)	328 (41.6%)	136 (17.3%)	32 (4.1%)	
**Facility characteristics**					
**Facility-type, n. (%)**					**<0.01**
Community	119 (38%)	90 (28.8%)	83 (26.5%)	21 (6.7%)	
Comprehensive	484 (36.9%)	389 (30.4%)	354 (27.7%)	50 (3.9%)	
Academic/Research	544 (35.3%)	741 (48.18%)	216 (14%)	40 (2.5%)	
Integrated cancer program	165 (47.1%)	96 (27.4%)	73 (20.8%)	16 (4.5%)	

Statistical analysis was performed using SPSS version 23. Baseline characteristics were compared using one-way ANOVA test with Post Hoc test for continuous variables, Pearson *chi-*square and likelihood ratio tests for categorical variables. We used multinomial logistic regression models to analyze differences between the treatment groups including significant variables in the univariable analysis. In the logistic regression analysis, we used (Radical Cystectomy and Chemo-Radiotherapy) group as the reference category. Then, we performed Kaplan-Meier survival analysis for all survival predictor variables. We also reported survival rates at 2 years and 5 years follow up. We categorized patients into two age groups with cutoff value of 66 years old which is the median age for the entire cohort. Pairwise survival analysis was also done between the treatment groups and was reported. Cox regression model was used to perform the univariable and multivariable survival analysis. We included all the significant survival predictors on Kaplan-Meier analysis in the Cox regression model. Indicator variables included female sex, score ‘≥2’ for the Charlson\Deyo comorbidity score, Integrated cancer program for the facility-type, cystectomy and chemo-radiotherapy for treatment and 7th edition for the TNM edition. Survival time was calculated using time from diagnosis to last contact or death (months) and the vital status (live or death) at last contact.

## Results

Final analysis included 3,481 patients. Most patients received either RC and chemotherapy or chemotherapy alone (1316 ‘37.8%’ and 1312 ‘38.7%’, respectively). There was a significant difference between the four groups regarding age (P < 0.01). Post Hoc testing revealed significant differences between pairs groups, with patients in ‘Chemo-radiotherapy’ group (69, SD 11.7) were significantly older than patients in each of the other three groups (chemotherapy only 67, SD 11, P < 0.01) (RC + chemotherapy 64, SD 9.7, P < 0.01) (RC + chemo-radiotherapy 64, SD 10.2, P < 0.01). Also, patients is ‘chemotherapy only’ group were significantly older than ‘RC + chemotherapy’ group (P < 0.01) and ‘RC + chemo-radiotherapy’ group (P = 0.02). There was no significant difference in age between ‘RC + chemotherapy’ and ‘RC + chemo-radiotherapy’ (P = 0.95). There was no difference among the four groups regarding male to female ratio (*P* = 0.43). White race to non-white race ratio was found to be statistically different among the groups, favoring receiving more invasive treatment for white patients (91.9% and 92% white patients in ‘RC + chemotherapy’ group and ‘RC + chemo-radiotherapy’ group respectively, vs 88.9% and 87.5% in chemotherapy only and chemo-radiotherapy) (*P* = 0.01). There was no significant difference among treatment groups regarding medical comorbidities that were categorized using Charlson-Deyo score (*P = *0.54).

Further analysis showed multiple social and demographic disparities among different treatment groups. Patient with private insurance were more likely to receive invasive treatment with chemotherapy and radical cystectomy (47.5%), while majority of patients with no insurance, Medicaid and Medicare were more likely to receive chemotherapy alone (43.2%, 39.8% and 39%, respectively) (*P* < 0.01). Patients who received their care at an academic/research facility were more likely to receive combination chemotherapy and radical cystectomy (48.08%) compared to those receiving care at community facilities (28.7%), comprehensive care facilities (30.4%), and integrated cancer facilities (27.4%) with these patients all more likely to receive chemotherapy alone (*P* < 0.01). There was no significant difference among treatment groups regarding different household income levels (*P* = 0.08) or education levels (*P* = 0.06).

Multivariable analysis comparing treatment groups was done using multinomial logistic regression model with (RC + Chemo-Radiotherapy) group as the reference category. We included significant variables in the univariable analysis in this model. Results are demonstrated in Table 2. The model showed that older age was a significant predictor for receiving chemotherapy only (OR 1.04, 95%CI 1.02–1.06, *P* < 0.01) and chemo-radiotherapy (OR 1.06, 95%CI 1.03–1.08, *P* < 0.01). Treatment in academic\research institutions was a predictor of receiving RC + chemotherapy (OR 3.15, 95%CI 1.69–5.87, *P* < 0.01).

**Table 2.** Multivariable multinomial logistic regression analysis.

Covariates		Chemotherapy only			RC + Chemotherapy			Chemo-radiotherapy		
		OR	95% CI	*P*	OR	95% CI	*P*	OR	95% CI	*P*
Age		**1.04**	**1.02–1.06**	**≤0.01**	1.01	0.99–1.03	0.37	**1.06**	**1.03–1.08**	**≤0.01**
sex (male)		1.29	0.87–1.92	0.2	1.24	0.83–1.84	0.29	1.12	0.74–1.68	0.59
Race (White)		0.66	0.34–1.3	0.23	1.06	0.53–2.12	0.86	0.5	0.25–1.01	0.05
Insurance status										
Uninsured		2.53	0.43–14.7	0.3	1.18	0.21–6.7	0.85	1.38	0.23–8.35	0.72
Private		2	0.43–9.29	0.37	1.69	0.37–7.63	0.49	0.99	0.21–4.69	0.99
Medicaid		1.97	0.38–10.1	0.42	0.97	0.19–4.87	0.97	1.33	0.25–6.99	0.73
Medicare		1.26	0.27–5.84	0.76	0.96	0.21–4.29	0.96	0.77	0.16–3.62	0.74
Other government		Ref								
Facility Type										
Community		0.56	0.28–1.12	0.1	0.72	0.35–1.47	0.37	0.89	0.43–1.84	0.75
Comprehensive		0.96	0.53–1.75	0.91	1.32	0.72–2.43	0.36	1.59	0.85–2.95	0.14
Academic\research		1.36	0.74–2.51	0.31	**3.15**	**1.69–5.87**	**≤0.01**	1.23	0.647–2.337	0.52
Integrated cancer program		Ref								

*The reference category is Radical Cystectomy and Chemo-Radiotherapy

**Figure 2.**
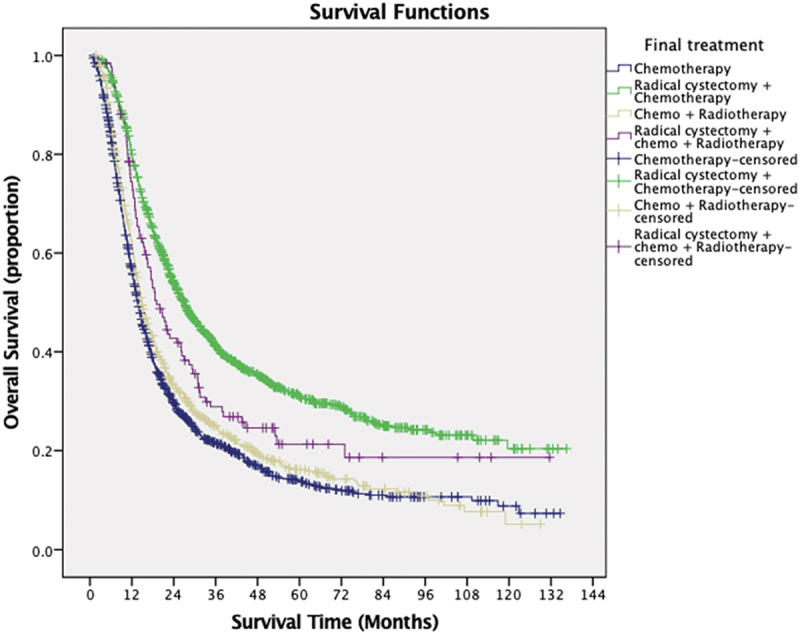
Kaplan-Meier curve demonstrating overall survival according to treatment.

The median follow-up period was 12.8 months (interquartile range, 5.8–26.7 months). The median overall survival of the cohort was 18 months. Kaplan-Meier survival analysis is demonstrated in Table 3. Our analysis showed that age less than 66 years old, male sex, lower Charlson\Deyo score, receiving treatment at academic centers or integrated cancer programs were significantly associated with longer median survival time. Patients with Medicaid and Medicare or no insurance showed significantly shorter median survival time than patients with private insurance or other governmental insurance. Patients with low household income (<38,000) showed significantly worse survival than patients with high income (>63,000). Patients who underwent combination therapy with chemotherapy and radical cystectomy were found to have the longest median survival time of 27 months with significantly higher 2-year survival rate (53.9%) and 5-year survival rate (30.9%). Patients undergoing chemoradiotherapy with radical cystectomy, chemotherapy alone, and chemoradiotherapy had median survival times of 19.1, 13.7, and 14.7 months, respectively. Kaplan- Meier curve is illustrated in Figure 2 (*P* < 0.01). Race and education status were not statistically significant predictors of survival. In view of different definitions of node-positive disease between 6th and 7th editions of TNM staging system, we compared the survival between patients with different editions of TNM staging. Patients with reported TNM staging using edition 7 showed statistically significant higher survival (Table 3).

**Table 3.** Kaplan-Meier survival analysis.

Variables	(No. of cases)		Median survival (months)	95% Confidence interval	2-year survival rate	5-year survival rate	P value
**Age**							**<0.01**
≤ 66 years old	1761		18.8	17.6–20.1	42.7%	24.7%	
>66 years old	1720		16.7	15.7–17.8	37.4%	17.3%	
**Sex**							**<0.01**
Male	2493		18.7	17.7–19.7	41.8%	21.9%	
Female	988		16.3	14.9–17.7	35.9%	18.6%	
**Race**							0.95
White	3129		18.1	17.3–19	40.2%	20.8%	
Non-white	352		16.3	13.8–18.8	39%	23.4%	
**Charlson\Deyo score**							**<0.01**
0	2614		18.8	17.7–19.9	41.9%	22.5%	
1	659		16.4	15–17.8	36.9%	17.4%	
≥2	208		14	12.7–15.3	26.4%	12.9%	
**Primary Payer**							**<0.01**
Uninsured	139		17.2	13.8–20.6	40.1%	24.9%	
Private	1178		20.4	18.6–22.2	45.1%	24.8%	
Medicaid	261		15.6	13.8–17.4	36.3%	19.8%	
Medicare	1857		17	15.9–18.1	37.2%	18.3%	
Other government	46		22.2	10.4–33.9	44.3%	28.5%	
**Median household income is**							**0.01**
<38,000	562		16.1	13.4–17.8	36.6%	20.2%	
38,000–47,999	897		18.6	17.1–20.2	40.7%	17.5%	
48,000–62,999	979		17.4	15.9–19	38.7%	19.7	
>63,000	1043		18.9	17.1–20.6	42.6%	25.6%	
**No. of high school diploma**							0.05
21%	546		19.5	17.1–21.9	41.7%	22.8%	
13% – 20.9%	919		16.5	15.3–17.7	36.9%	18.4%	
7% – 12.9%	1228		18.3	16.9–19.6	40.9%	21.9%	
<7%	788		18.6	16.7–20.4	41.5%	21.6%	
**Facility-type**							**<0.01**
Community	313		16.5	14.1–18.9	38.9%	18.1%	
Comprehensive	1277		15.6	14.5–16.7	33.6%	16.4%	
Academic/Research	1541		20.4	18.8–22.1	45.1%	24.8%	
Integrated cancer program	350		19.5	15.8–23.2	42.5%	23.9%	
**Treatment group**							**<0.01**
Chemotherapy only	1312		13.7	12.9–14.5	29.6%	13.8%	
RC + Chemotherapy	1316		27	24.7–29.3	53.9%	30.9%	
Chemo-Radiotherapy	726		14.7	13.5–16	33.1%	15.8%	
RC + Chemo-Radiotherapy	127		19.1	15.7–22.4	41.8%	21.3%	
**TNM staging edition**							**0.02**
Edition 6	1625		17.2	16.1–18.2	38.2%	20%	
Edition 7	1856		18.8	17.5–20.1	41.9%	22.5%

Multivariable analysis was done using a cox proportional hazard regression model. It showed that age (*P* < 0.01), sex (*P* < 0.01), Charlson\Deyo score (*P* < 0.01), final treatment (*P* < 0.01), facility-type (*P* < 0.01) and TNM edition (*P* 0.01) were independent predictors of overall survival. Higher age was associated with worse survival with HR 1.008 (95%CI 1.004–1.011) (*P* < 0.01). Male sex was an independent predictor of better survival with HR 0.83 (95% CI 0.76–0.9) (*P* < 0.01). When compared to Charlson\Deyo score ‘≥2’, scores ‘0’ and ‘1’ were independent predictors of better survival, with HR 0.69 (95%CI 0.95–0.82, *P* < 0.01) and 0.83 (0.69–0.98, *P* 0.03), respectively. Comprehensive community facility was an independent predictor of worse survival with HR 1.26 (95% CI 1.09–1.45) (*P* < 0.01). Radical cystectomy and chemotherapy were found to be significantly associated with longer overall survival with HR 0.79 (95%CI 0.64–0.98) (*P* 0.03), while chemotherapy alone (HR 1.46, 95%CI 1.18–1.81, *P* < 0.01) and Chemo-radiotherapy (HR 1.25, 95%CI 1.01–1.56, *P* 0.04) were associated with worse overall survival. Results of Cox-regression analysis are demonstrated in Table 4.

**Table 4.** Cox Regression multivariable survival analysis.

Variables	Adjusted HR	95% CI	P value
**Age**	1.008	1.004–1.011	**<0.01**
**Sex**			
Male	0.83	0.76–0.9	**<0.01**
Female	Ref.		
**Charlson\Deyo score**			**<0.01**
0	0.69	0.59–0.82	**<0.01**
1	0.83	0.69–0.98	**0.03**
≥2	Ref.		
**Facility-type**			**<0.01**
Community	1.15	0.96–1.37	0.12
Comprehensive	1.26	1.09–1.45	**0.01**
Academic/Research	1.04	0.9–1.2	0.55
Integrated cancer program	Ref.		
**Treatment group**			**<0.01**
Chemotherapy only	1.46	1.18–1.81	**<0.01**
RC + Chemotherapy	0.79	0.64–0.98	**0.03**
Chemo-Radiotherapy	1.25	1.01–1.56	**0.04**
RC + Chemo-Radiotherapy	Ref.		
**TNM Edition**			
6^th^ edition	1.11	1.02–1.2	**0.01**
7^th^ edition	Ref.		

Pairwise analysis was also done between the treatment groups (Table 5) and Patients who received RC + chemotherapy were found to have significantly longer survival than chemotherapy only (P < 0.01), chemo-radiotherapy (P < 0.01) and RC + chemo-radiotherapy (P = 0.01). Also, RC + chemo-radiotherapy showed longer survival than chemotherapy only (P = 0.01) and chemo-radiotherapy (P = 0.01). Chemotherapy only and chemo-radiotherapy were similar (P = 0.05).

**Table 5.** Pairwise survival comparison between treatment groups.

Treatment groups	P value
Chemotherapy only vs. RC ± Chemotherapy	**<0.01**
Chemotherapy only vs. RC ± Chemo-radiotherapy	**0.01**
Chemotherapy only vs. Chemo-radiotherapy	0.05
Chemo-radiotherapy vs. RC ± Chemotherapy	**<0.01**
Chemo-radiotherapy vs. RC ± Chemo-radiotherapy	**0.01**
RC ± Chemotherapy vs. RC ± Chemo-radiotherapy	**0.01**

## Discussion

Patients with cN+ disease were historically categorized with patients demonstrating distant metastatic disease. As a result, these patients frequently were treated with palliative chemotherapy only. More recently, there has been evidence surfacing that cN+ patients undergoing NAC and RC achieve improved long-term survival when compared to palliative chemotherapy only. Specifically, large retrospective analyses of national cancer registries have been performed both in the Czech Republic and the United States [5,7].

Stanik et al [7] demonstrated a 21% reduced risk of mortality among patients with cN+ disease undergoing chemotherapy and RC compared to chemotherapy alone in their cohort of 661 patients. Likewise, Galsky et al [5] demonstrated that there is a survival benefit in patients receiving chemotherapy, both in the neoadjuvant and adjuvant settings, and RC when compared to chemotherapy alone. Their data analysis also demonstrated that in terms of overall survival, those treated with RC alone fared better than chemotherapy alone. Both of these analyses prove valuable in dispelling the notion that cN+ patients should be approached from a palliative standpoint, however, they do not include patients who underwent radiotherapy as a component of their management.

Our analysis of the NCDB registry of cN+ patients echoes these prior analyses by demonstrating longest overall survival among patients undergoing chemotherapy in combination with RC. Radiotherapy proves to have a significant role in management of cN+ patients as demonstrated by our analysis that revealed nearly 25% of cN+ received radiotherapy as a part of their treatment. Despite its frequent use in this patient population, the addition of radiotherapy to chemotherapy demonstrated a one month advantage in terms of overall survival in this cohort. While our data analysis did not reveal any significant difference between chemotherapy alone and combination chemotherapy and radiation therapy, it is worth noting that historically radiation therapy was limited to targeting the bladder, excluding the pelvic lymph nodes, due to fear of toxicity to the surrounding abdominal structures [9]. Also, in our multivariable analysis, adding radiotherapy did not show association with improved overall survival. Only the combination of radical cystectomy and chemotherapy predicted longer overall survival. These results suggest that adding radiotherapy has no benefit in this group of patients.

The feasibility of radiation therapy in cN+ patients was recently addressed in a small prospective study of 38 patients by Huddart et al [9]. In their patient cohort median overall survival for cN+ patients was 1.9 years following radiation therapy. 81.6% of these patients underwent neoadjuvant chemotherapy. The findings of their study support our findings that the optimal treatment in terms of survival benefit for cN+ patients remain neoadjuvant chemotherapy and RC. However, this finding is not to overshadow the fact that some patients are not surgical candidates and radiation therapy including the pelvic nodes may provide a reasonable alternative in this population, albeit at the expense of more frequent local surveillance given the high rate of local recurrence identified in the prospective cohort studied by Huddart et al (31.4% developed a muscle invasive recurrence and 20% developed superficial recurrence) [5]. This concept will require continued future investigation.

Not surprisingly, certain socioeconomic and patient characteristics influenced treatment decisions. Patients who were younger were significantly more likely to receive definitive surgical treatment with chemotherapy or chemoradiotherapy, while those who were older were more likely to receive chemotherapy only or chemoradiotherapy. Patients receiving treatment at an academic\research center were more likely to undergo RC and chemotherapy. Of these, only age and facility-type were found to impact overall survival with race and insurance status not impacting survival. On the other hand, certain factors that were not associated with treatment received were associated with survival. Male patients and patients with less co-morbidities were found to have increased overall survival. Patients classified based on 6th edition of TNM staging had worse overall survival than those classified based on the 7th edition despite the 7th edition including more advanced disease including the iliac nodes. We believe that this finding is related to time of treatment and advances in the understanding of optimal treatment in this patient population, although a clear explanation is difficult to reach based on the available dataset.

Our study was limited in several ways both due to the retrospective nature of data collection as well as limited access to specific information provided by the NCDB. For example, chemotherapy regimens are classified according to whether they are single or multi-agent, but information regarding specific drug regimen, dose, and duration are not readily obtained. Likewise, dose of radiation, timing, and field are not readily ascertained. Additionally, the NCDB does not provide data on cancer-specific mortality which leads us to use all-cause mortality as our primary endpoint. Also, data on tumor histology and histological variants are limited in the database. Despite the limitations of the study the authors believe it adds to the literature as it represents, to our knowledge, the only large dataset of cN+ patients that includes patients who underwent radiotherapy.

## Conclusion

The combination of chemotherapy and radical cystectomy appears to confer a statistically significant survival benefit in cN+ patients. The addition of radiation therapy to chemotherapy did not improve survival compared to chemotherapy alone in this patient cohort from the NCDB. Predictors of improved overall survival in this cohort include male sex, younger age, less co-morbidities, and treatment with RC and chemotherapy.
